# Runx1 Is Critical for PTH-induced Onset of Mesenchymal Progenitor Cell Chondrogenic Differentiation

**DOI:** 10.1371/journal.pone.0074255

**Published:** 2013-09-18

**Authors:** Jinwu Wang, Xudong Wang, Jonathan D. Holz, Timothy Rutkowski, Yongjun Wang, Zhenan Zhu, Yufeng Dong

**Affiliations:** 1 Department of Orthopaedics, Ninth People’s Hospital, Shanghai Jiao Tong University School of Medicine, Shanghai, China; 2 Department of Oral and Maxillofacial Surgery, Ninth People’s Hospital, Shanghai Jiao Tong University School of Medicine, Shanghai Key Laboratory of Stomatology, Shanghai, China; 3 Department of Math and Natural Sciences, D’Youville College, Buffalo, New York, United States of America; 4 Center for Musculoskeletal Research, Department of Orthopaedics and Rehabilitation, University of Rochester School of Medicine, Rochester, New York, United States of America; 5 Institute of Spine, Longhua Hospital, Shanghai University of Traditional Chinese Medicine, Shanghai, China; University of Maryland School of Medicine, United States of America

## Abstract

Parathyroid hormone (PTH) plays a critical role in the regulation of chondrogenesis. In this study, we have found for the first time that Runt-related transcription factor 1 (Runx1) contributes to PTH-induced chondrogenesis. Upon PTH treatment, limb bud mesenchymal progenitor cells in micromass culture showed an enhanced chondrogenesis, which was associated with a significant increase of chondrogenic marker gene expression, such as type II collagen and type X collagen. Runx1 was also exclusively expressed in cells treated with PTH at the onset stage of chondrogenesis. Knockdown of Runx1 completely blunted PTH-mediated chondrogenesis. Furthermore, PTH induced *Runx1* expression and chondrogenesis were markedly reduced by inhibition of protein kinase A (PKA) signaling. Taken together, our present study indicates that chondrogenesis induced by PTH in mesenchymal progenitor cells is mediated by Runx1, which involves the activation of PKA. These data provide a novel insight into understanding the molecular mechanisms behind PTH-enhanced cartilage regeneration.

## Introduction

Osteoarthritis (OA) is a degenerative joint disease that involves the destruction of articular cartilage and is the leading cause of disability in the U.S. Molecules that promote the selective differentiation of multipotent Mesenchymal Progenitor Cells (MPCs) into chondrocytes constitute a class of putative therapeutic agents, potentially capable of stimulating cartilage repair in OA patients.

Parathyroid hormone (PTH) is a major systemic regulator of calcium and phosphate homeostasis in bone [1]–[3]. Although PTH is naturally produced from the parathyroid gland as an 84 amino-acid peptide (1–84), it has been determined that the N-terminal fragment, PTH1-34 (Teriparatide), can reproduce the majority of the biological actions attributed to full-length PTH [4]. Many studies showed that continuous exposure to PTH leads to hypercalcemia and a net decrease in bone volume, which is referred to as its ‘catabolic effect’. In contrast, intermittent (once daily) exogenous PTH administration has an anabolic effect on bone [5], [6]. Since Teriparatide has been approved by US Food and Drug Administration (FDA) to treat osteoporosis [7], its potential use in clinical bone and cartilage regeneration has become a hot topic. In 1999 Andreassen *et al.* were the first to report the efficacy of intermittent PTH1–34 therapies on rat tibia fracture healing [8]. Additionally, a number of studies have also shown that PTH1–34 enhances bone repair regardless of the skeletal site [9]. These studies suggest that PTH1–34 plays a role not only in bone remodeling, but also in modulation of osteogenesis during skeletal repair. In contrast, Kakar et al showed that PTH1–34 preferentially enhanced chondrogenesis over osteogenesis by regulating Indian hedgehog (Ihh) signaling in their mouse closed femoral fracture model [10]. This enhanced chondrogenesis leads to increased cartilaginous callus formation in the early phase of fracture repair; subsequently PTH1–34 enhances chondrocyte maturation and mineralization in the fracture callus, as evidenced by both an earlier peak in Sox9 expression and the corresponding earlier induction of type X collagen. Although several mechanisms have been postulated for this observation, the exact mechanisms for PTH-induced chondrogenesis *in vivo* remain unclear.

Runt-related transcription factor 1 (Runx1) belongs to a small family of transcription factors, including Runx1, Runx2 and Runx3, and is composed of an NH2-terminal DNA-binding runt homology domain followed by a transcriptional activation domain and COOH-terminal negative regulatory domain [11]. Runx1 has been initially identified at a breakpoint of human chromosome 21q22 in the t (8; 21) translocation, which is required for proper hematopoiesis [12], [13]. Although numerous studies with respect to Runx1 have focused largely on its functional significance in the hematopoietic system, it has been shown that Runx1 and Runx2 expression overlap during chondrogenesis, suggesting a cooperative and not redundant function of these two factors in chondrogenesis [14], [15]. Furthermore, a recent study showed that Runx1 and Runx2 cooperatively regulate sternal morphogenesis and the commitment of MPCs to become chondrocytes through the induction of *Sox5* and *Sox6* [16]. Since both PTH and Runx1 are strongly related to early chondrogenesis, we therefore speculate that PTH induces MPC differentiation to the chondrocyte lineage through up-regulation of *Runx1*.

The Protein Kinase A (PKA) pathway has been studied extensively in osteoblasts [5], [17]. PKA consists of two regulatory subunits and two catalytic subunits and is localized in the cytosol as an inactive enzyme. PKA is activated when cyclic 3′, 5′-adenosine monophosphate (cAMP) binds to each of the regulatory subunits. The catalytic subunits are then released, allowing them to translocate to the nucleus and activate gene expression by phosphorylating the cAMP response element (CRE)-binding protein (CREB) at Ser-133 [18], [19]. Although PKA signaling is known to play a critical role in osteogenesis of MPCs, the involvement of PKA in PTH-induced chondrogenesis still remains undefined.

In this study, primary limb bud derived MPCs isolated from mouse embryos were cultured in high-density micromass creating a chondrogenic environment [15]. PTH peptide fragment 1–34 was administered intermittently to the cultured MPCs. Chondrogenic markers were then analyzed to study the effects of PTH administration on chondrogenesis during MPC micromass culture. Our results demonstrate that activation of PKA signaling and *Runx1* are required for the PTH-induced commitment of MPC to the chondrocyte lineage.

## Materials and Methods

### Isolation and Culture of Limb Bud MPCs

Embryos were harvested from CD1 pregnant mice at stage embryonic day 11.5 (E11.5) with approval from the animal medical ethics committees of Shanghai Jiaotong University. Briefly, pregnant mice were sacrificed by CO_2_ and followed by cervical dislocation. After removal of the uterus, embryos were isolated using a dissecting microscope (Olympus SZX12) and rinsed with sterile ice-cold Phosphate Buffered Saline (PBS). Limb bud-derived MPCs were further isolated as previously described [14]. Briefly, forelimbs were digested with 1 U/ml dispase for 3–4 hours at 37°C with continuous rotation at 70 rpm using a reciprocal shaking bath. Cells were then filtered through a 40-µm strainer before being re-suspended in 40% DMEM/60% F12 media supplemented with 10% Fetal bovine serum (FBS) and antibiotics. Cells were seeded in micromass at a high-density of 1×10^5^ cells per 10 µl of media in 12-well plates. The cells were maintained in culture for 24 hours before being treated. Samples were collected as described at various time points (6 hours, 12 hours, 3, 5, and 7 days).

### PTH Treatment

According to the PTH administration methods [20], limb bud cells were divided into two groups: the control group (Vehicle Dimethyl Sulfoxide [DMSO] treatment), and the intermittent PTH treatment group. After 24 hours of micromass culture, part of the limb bud cells were stimulated for the first 6 hours with or without (Vehicle control group) 10^−8^ M recombinant human PTH1-34 (Sigma, Inc) before harvested as day 1 samples for Real time RT-PCR and 6 hour samples for western blot. After 6 hours of stimulation, the culture medium in the PTH group was changed to fresh regular culture medium without PTH. The PTH administration cycle was repeated every 48 hours until samples were harvested at day 3, 5, and 7.

### Chondrocyte Nodule Detection

Alcian blue staining was used to detect chondrocyte nodule formation after 3, 5, and 7 days of culture. Cells in micromass culture were rinsed with PBS and fixed in 10% formaldehyde in PBS for 20 minutes. Cultures were washed with water three times and stained in 1% Alcian blue in 3% glacial acetic acid for 24 hours. Cultures were de-stained in 70% ethanol two times and stored in water for image capture. Samples were then quantified by solubilizing in 6 M guanidine hydrochloride for 8 hours at room temperature and absorbance was measured using a spectrophotometer at 620 nm as previously described [21].

### Immunofluorescence Analysis

For immunofluorescence, limb bud cells were plated at 1,000 cells/cm^2^ on coverslips and grown for 24 hours in medium. When at least 60% of confluence was reached, cells were serum deprived for 24 hours and treated with PTH for 6 hours. Cells were then fixed in 4% paraformaldehyde in PBS for 20 minutes at room temperature and permeabilized with 0.3% Triton X-100 in PBS for 30 minutes. Cells were washed in PBS and incubated with 0.5% Bovine serum albumin (BSA) dissolved in PBS at room temperature for 20 minutes. Cultures were then incubated for 2 hours at room temperature with the following primary antibodies: rabbit anti-Runx1 (Santa Cruz Biotechnology) diluted 1∶50 in PBS. After washing with PBS, cells were incubated with FITC conjugated anti-rabbit IgG for 1 hour at room temperature. Reaction controls were performed using a non-immune rabbit immunoglobulin IgG, or by omitting the primary antibody. Cover slips were mounted on slides with PBS/glycerol (1∶1). Slides were imaged using fluorescent microscopy on Zeiss Axioplan microscopy.

### RNA Interference Experiments

Limb bud cell suspensions (2×10^7^/ml) were transduced with either Runx1 shRNA lentiviral Transduction Particles or pLKO.1-puro Non-Target shRNA Control Transduction Particles. Runx1 shRNA lentiviral Transduction Particles contained Runx1 short hairpin 5′CCGGGCCCTCCTACCATCTATACTACTCGAGTAGTATAGATGGTAGGAGGGCTTTTTG3’ cloned to lentivirus vector pLKO.1 (Sigma, Gene ID 12394). In both, the ratio of 500 particles/cell was used in growth media for 6 hours. Infected cells were then seeded in micromass and maintained in growth media for 24 hours before PTH treatment. After 4 days of the PTH treatment cycle, Alcian blue staining and real-time PCR were performed as described.

### Inhibition of PKA Signaling

The cells were maintained in micromass culture for 24 hours before being treated with H-89 (Dihydrochloride), a selective PKA inhibitor purchased from Cell Signaling Technology. 10 µM H-89 was added to culture daily. After 4 days of treatment, samples were collected and prepared as described.

### Western Blot Analysis

40 µg of total protein from different samples was used for Western blot analysis. The samples were separated by 10–15% SDS–PAGE gel. These proteins were then transferred to polyvinylidene difluoride (PVDF, Millipore) membranes. After blocking with 5% non-fat milk, the PVDF membranes were incubated with primary antibodies in a Tris-buffered saline (TBS) buffer overnight at 4°C. These primary antibodies were anti-Runx1 (Santa Cruz Biotechnology) or anti-Phospho-PKA Thr197 (Cell Signaling Technology). On the following day, PVDF membranes were incubated with appropriate secondary antibodies for 2 hours at room temperature. After the membranes had been soaked in an enhanced chemiluminescence reagent (Thermo Scientific) for 5 minutes, the blots were visualized using X-ray film. β-actin antibody was used as a loading control. Quantitative band-intensity analysis of Western blots was performed using ImageJ software and normalized to β-actin.

### Real-time PCR Analysis

Total RNA was isolated from micromass culture using RNeasy Mini Kit from Qiagen. Inc. One microgram of RNA was subjected to reverse transcription using the iScript cDNA synthesis Kit (Bio-Rad). The cDNA was then amplified via real-time PCR using an ABI 7500 Real-time PCR System (Applied Biosystems) and SYBR® Green Real time PCR Supermix (Bio-Rad). The primers used for real-time PCR are listed in Table 1, and β-actin was used as the housekeeping gene. Quantification of the relative expression levels of these target genes was achieved by normalizing to β-actin using the ΔΔCt method.

**Table 1:** Mouse Gene Primers Used for Real-Time RT-PCR Experiments.

	Forward primer	Reverse primer	Accession number
Runx1	GCATGGTGGAGGTACTAGCTG	GCCGTCCACTGTGATTTTG	NM009821
Col2a1	ACTGGTAAGTGGGGCAAGAC	CCACACCAAATTCCTGTTCA	BC052326
Sox9	AGGAAGCTGGCAGACCAGTA	CGTTCTTCACCGACT TCCTC	AF421878
Col10a1	CTTTGTGTGCCTTTCAATCG	GTGAGGTACAGCCTACCAGTTTT	X67348
β-actin	AGATGTGGATCAGCAAGCAG	GCGCAAGTTAGGTTTTGTCA	NM007393

### Cell Viability Assay

Cells in micromass cultures at day 5 were trypsinized using 0.25% trypsin-EDTA (Invitrogen), then counted, and stained by 0.4% trypan blue solution. Cell viability was estimated by cell counting via hemocytometer in which non-viable cells are blue, viable cells are unstained.

### Statistical Analysis

All the above experiments were repeated at least three times independently. All the data was presented as mean ± SD. Statistical significance among the groups was assessed with one-way ANOVA. The level of significance was *P*<0.05.

## Results

### Chondrogenic Differentiation of Limb Bud MPCs is Promoted by Intermittent PTH Treatment

**Figure 1:**
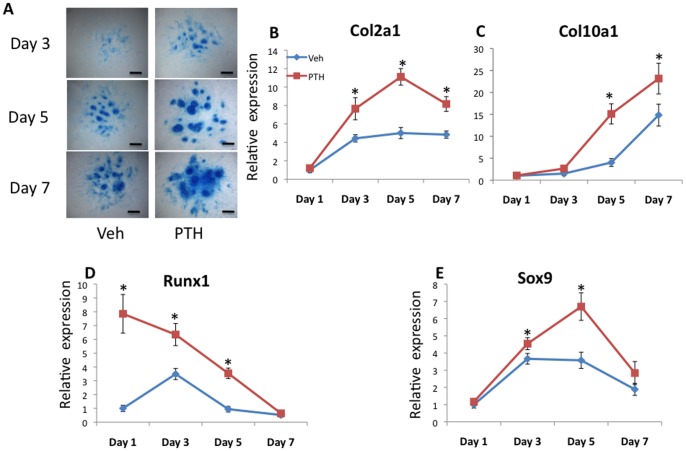
PTH promotes chondrogenesis in limb bud cell micromass culture. Limb bud cells were cultured in micromass and treated intermittently with 10^−8^M PTH 1–34 before being harvested for Alcian blue staining and RT-PCR analysis. (A) PTH treatment resulted in increased cartilage nodule formation at days 3, 5, and 7, compared to vehicle-treated controls. Scale bars, 100 µm. (B, C, D and E) PTH treatment increased Col2α1 expression at days 3, 5, and 7, with maximal increase at day 5. Col10a1 expression was increased at days 5 and 7. Runx1 expression was significantly increased by PTH at days 1, 3 and 5 with maximal increase seen at day 1. Sox9 levels were increased at days 3 and 5. Data are means ± SD of three independent experiments performed in duplicate and the control gene expression level at day 3 was set at 1. (*p<0.05 compared with control at same time point).

To analyze the effects of intermittent PTH administration methods on MPC chondrogenic differentiation, high-density limb bud cell micromass culture was used in this study. Under these conditions, the initial appearance of cartilage matrix and chondrocytes occurred on day 3 of culture and progressed over the next 4 days as shown via enhanced number and size of chondrogenic nodules (Fig. 1A). By days 5 and 7, strong Alcian blue staining of cartilage matrix was observed inside the chondrogenic nodules (Fig. 1A). Meanwhile, PTH treatment accelerated formation of chondrogenic nodule at day 3, and the maximal increase in nodule formation by PTH was detected between days 5 and 7 (Fig. 1A). These findings indicate that PTH promotes chondrogenic differentiation in MPCs.

The mRNA expression levels of type II collagen (*Col2a1*), a marker of chondrogenic differentiation, and type X collagen (*Col10a1*), a marker of chondrocyte hypertrophy, were detected by real-time PCR. Consistent with Alcian blue staining, *Col2a1* mRNA expression in PTH treated groups was significantly up regulated during days 3–7, compared to vehicle-treated controls (Fig. 1B). *Col10a1* expression in vehicle control cells rapidly increased from day 5 to day 7, indicating chondrocytes started to undergo maturation and hypertrophy at this stage (Fig. 1C). Moreover, PTH significantly induced *Col10a1* expression at day 5 and 7. These data confirmed that PTH rapidly induces differentiation of MPCs into chondrocytes in micromass culture *in vitro*.

To further uncover the possible molecules responsible for PTH-induced chondrogenesis during limb bud MPC chondrogenic differentiation, mRNA expression of *Sox9* and *Runx1*, two important regulators of early chondrogenesis, were quantified using RT-PCR. As shown in Fig. 1D, control cells initially showed a 3-fold increase in *Runx1* expression during days 1–3, which represents the onset stage of chondrogenesis, and started to decrease at day 5 and day 7, which represents the stage of chondrocyte maturation and hypertrophy. This result is consistent with our previous findings [14], which suggest that Runx1 functions mainly at the onset stage of chondrogenesis. Interestingly, PTH-treated cells showed a rapid, significant increase (8-fold) in *Runx1* expression relative to controls at day 1 after 6 hours treatment with PTH, but this effect diminished at days 3 and 5, and returned to levels not significantly different from controls at day 7 when chondrocytes became hypertrophic (Fig. 1D). Surprisingly, the expression levels of Sox9 in PTH-treated cells were not significantly different from that observed in control cells at day 1 and showed only a moderate increase at day 3 with a maximal increase at day 5 (Fig. 1E). When compared to *Runx1* expression, this delayed increase in *Sox9* gene expression implies differential roles of the two genes in PTH-induced chondrocyte nodule formation. Therefore, we hypothesize that PTH-induced chondrocyte nodule formation may function directly through the up-regulation of transcription factor *Runx1*, not *Sox9* at the onset stage of chondrogenesis.

### PTH Induces Runx1 Protein Expression in Limb Bud MPC Culture

**Figure 2:**
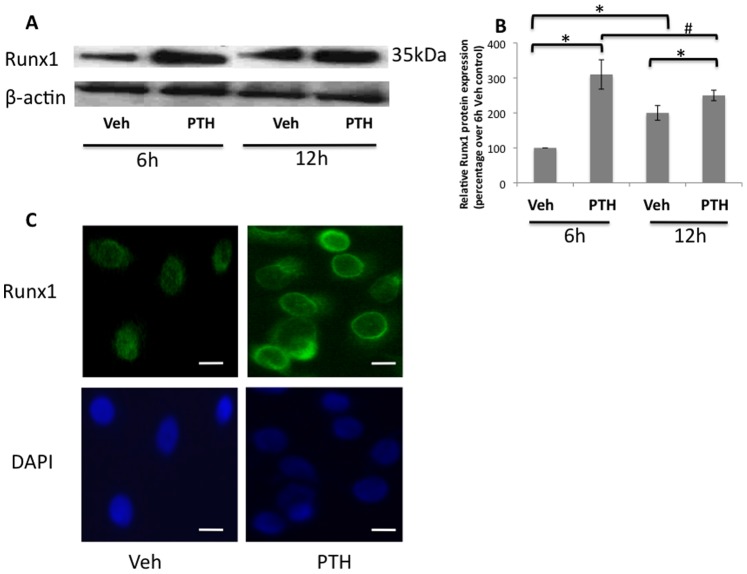
Up-regulation of Runx1 expression by PTH in limb bud mesenchymal progenitor cells. (A) Western blot analysis reveals that 6 hours and 12 hours of PTH treatment increased Runx1 protein levels in the limb bud cells, compared to DMSO (Vehicle control). β-actin was used as a loading control.(B) Quantification of Runx1 protein expression in Western blots. Data are means ± SD of three independent experiments. (*p<0.05 compared with control at same time point, #p<0.05 compared with PTH at 6 h). (C) In vehicle-treated controls endogenous Runx1 is localized to nuclear areas. Markedly increased peri-nuclear and nuclear labeling of Runx1 was visible in limb bud cells after treatment with PTH for 6 hours. Cells were counterstained with DAPI (blue). Scale bars, 5 µm.

To further study whether the expression of *Runx1* could be regulated in response to PTH treatment, we first analyzed Runx1 protein expression in MPCs. Western blot results show the protein levels of Runx1 in control cells were detectable at 6 hours in micromass culture, and increased slightly at 12 hours. PTH treatment significantly enhanced Runx1 expression both at 6 and 12 hours when compared with vehicle treated controls (Fig. 2A). Quantification of Runx1 expression in Western blots was also performed (Fig. 2B) using ImageJ software. These data suggest that PTH-induced rapid expression of *Runx1* was also reflected in the protein levels.

To confirm this rapid regulation of *Runx1* by PTH in MPCs, we performed immunofluorescence using Runx1 antibody in limb bud cells after 6 hours PTH treatment. Fig. 2C shows that most of the endogenous Runx1 localized to the nuclear area, while enhanced Runx1 labeling in both peri-nuclear and nuclear areas was observed as early as 6 hours after PTH treatment, when compared to controls. This finding suggests the possibility of direct regulation of *Runx1* by PTH during the chondrogenic differentiation of limb bud MPCs.

### Knockdown of Runx1 Impairs PTH-induced Chondrogenesis in MPC Culture

**Figure 3:**
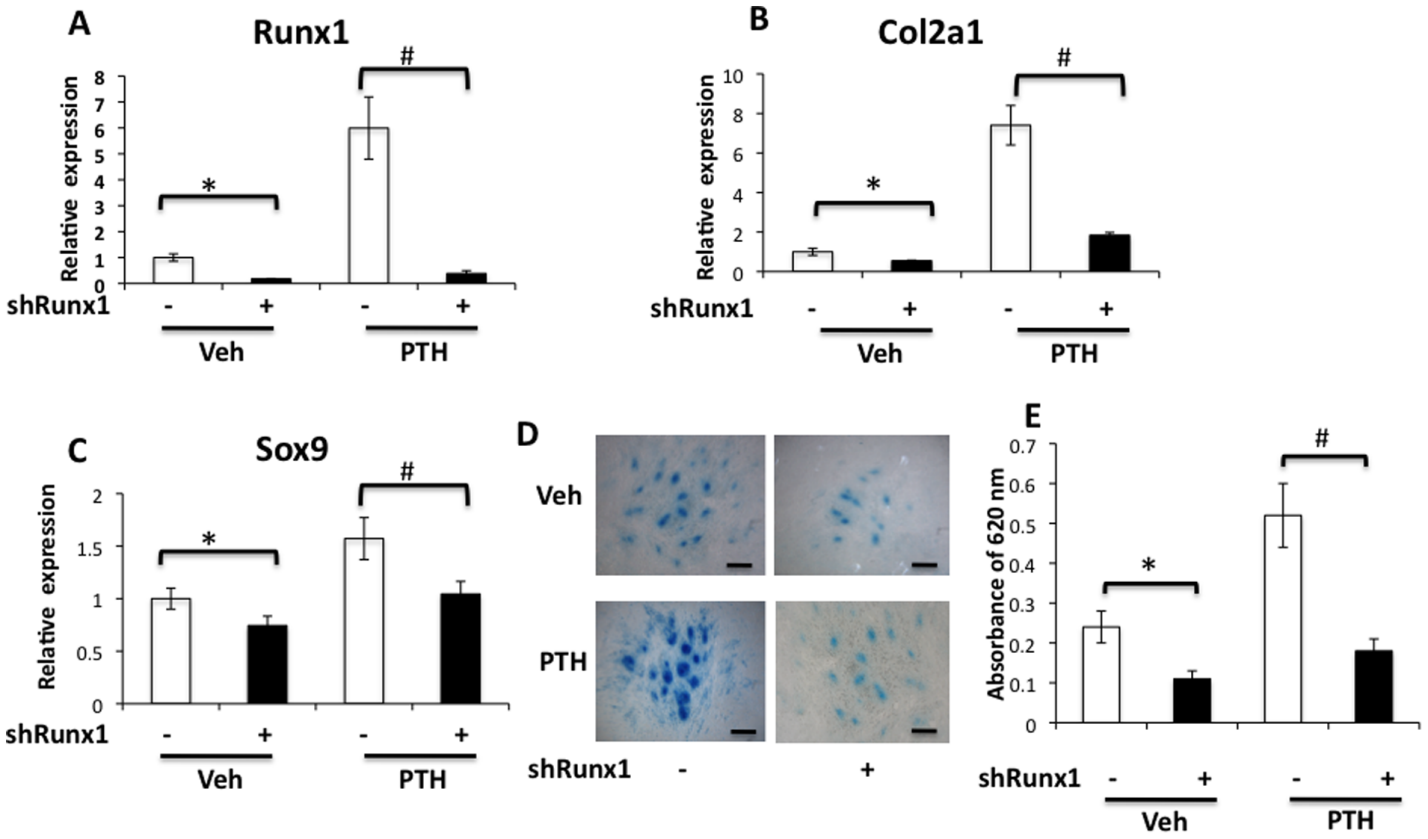
Knockdown of Runx1 impairs PTH-induced chondrogenesis. Limb bud cells were infected with Runx1 shRNA lentivirus and treated intermittently with PTH 1–34 for 4 days before being harvested at day 5 of culture for Alcian blue staining and RT-PCR analysis. (A) Runx1 knockdown significantly inhibited Runx1 gene expression and resulted in the inability of PTH to induce Runx1 expression. (B) Runx1 knockdown significantly inhibited type II collagen gene expression and significantly reduced PTH-induced type II collagen expression. (C) Sox9 gene expression was reduced by Runx1 knockdown in PTH- and vehicle-treated cells. (D) A drastic reduction in chondrocyte nodule formation was seen in PTH-treated cells that had Runx1 knocked down. Scale bars, 100 µm. (E) After solubilization in 6 M guanidine hydrochloride, blue staining taken up by the micromasses was quantified by measuring absorbance at 620 nm. Data (A, B, C, E) are means ± SD of three independent experiments performed in duplicate and the vehicle control gene expression level was set at 1. (*p<0.05 compared with vehicle control; #p<0.05 compared with PTH without shRunx1).

In order to elucidate the specific contribution of *Runx1* to the PTH-induced chondrogenic differentiation in limb bud MPCs, we measured changes in chondrogenic differentiation following knockdown of *Runx1* using an shRNA interference method. Fig. 3A shows an 80% decrease of *Runx1* gene expression 4 days after infection with *Runx1* shRNA lentivirus when compared to controls infected with non-target shRNA. Similar inhibition was also observed in PTH-treated MPCs indicating a high knockdown efficiency was achieved in our experiments (Fig. 3A). As previously mentioned, quantitative real-time PCR analysis revealed that PTH strongly increased Col2a1 expression in control MPCs; however, PTH was unable to induce Col2a1 expression in MPCs in the presence of Runx1 shRNA. These data indicate that *Runx1* may be required for PTH-induced cartilage matrix formation (Fig. 3B). Interestingly, knockdown of *Runx1* only mildly decreased *Sox9* expression levels in vehicle treated and PTH treated cells (Fig. 3C) suggesting only a partial regulation of *Sox9* by *Runx1* at this stage.

To further confirm the effect of knocking down *Runx1* on chondrogenic nodule formation in limb bud cell micromass culture, Alcian blue staining was also performed. As shown in Fig. 3D, fewer chondrogenic nodules were formed in *Runx1*-knockdown cells than in the shRNA control groups. Additionally, knock down of Runx1 using shRNA blunted PTH-induced chondrogenic nodule formation. Quantitative analysis of the absorbance of dye extracted from the nodules further confirmed a significant reduction in Alcian blue staining (Fig. 3E). These results support the conclusion that Runx1 is required for PTH-induced chondrogenesis during limb bud MPC differentiation.

### PKA Signaling is Involved in PTH-induced Runx1 Expression and Chondrogenesis in Limb Bud MPC Micromass Culture

**Figure 4:**
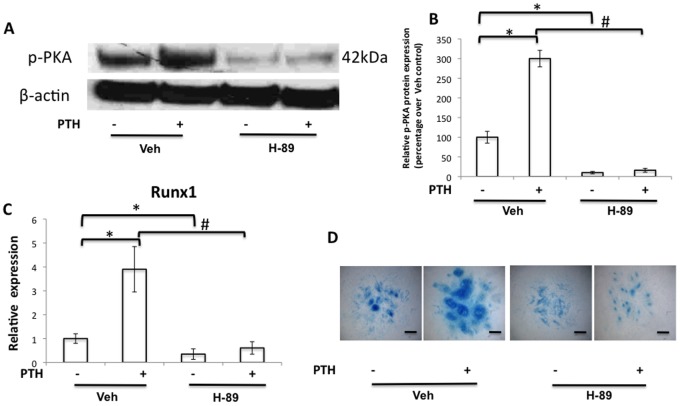
PKA inhibitor blocked the synthesis of Runx1 and chondrogenesis induced by PTH. Limb bud cells were treated for 4 days with 10 µM PKA inhibitor H-89 and/or PTH 1–34 before protein and RNA were extracted and Alcian blue staining at day 5 of culture. (A) Phospho-PKA (p-PKA) protein levels were visibly reduced by H-89. (B) Quantification of Phospho-PKA protein expression in Western blots using ImageJ software. Data are means ± SD of three independent experiments. (C) PTH-induced Runx1 expression was drastically reduced by co-treatment with H-89. Data are means ± SD of three independent experiments performed in duplicate and the control Runx1 expression level was set at 1. (D) A reduction in chondrocyte nodule formation was seen in H-89-treated cells with/without PTH. Scale bars, 100 µm. (*p<0.05 compared with vehicle control; #p<0.05 compared with PTH without H-89).

Previous studies have shown that many signaling molecules, such as PKA, protein kinase c (PKC), mitogen-activated protein kinases (MAPK), and Wnt signaling, are involved in various type of cell survival and differentiation that are regulated by PTH [22]–[28]. Most investigators believe that PKA activation and phosphorylation of cyclic AMP responsive element binding protein (CREB) are the major steps that mediate the anabolic effect of PTH *in vivo* [5]; here we focused only on PKA signaling. To determine whether inhibition of PKA signaling is sufficient to interfere with PTH-induced chondrogenesis, we examined the effect of H-89, an inhibitor of PKA activity, on PTH-mediated *Runx1* expression and chondrogenesis. After 4 days of treatment with PTH, as expected, we observed a significant increase (3-fold) in cellular expression of p-PKA when compared to control vehicle-treated cells. Co-treatment with H-89 decreased cellular expression of p-PKA to a similar level in both vehicle- and PTH-treated cells (Fig. 4A and 4B). This indicates that PKA signaling was significantly inhibited in these cells. Consistent with results observed in Fig. 1D, our Real time PCR data showed that PTH significantly increased *Runx1* expression in cells not treated with inhibitor, and H-89 treatment decreased Runx1 expression in vehicle-treated cells. In contrast, co-treatment with H-89 and PTH abolished the up-regulatory effects of PTH on *Runx1* expression (Fig. 4C). Furthermore, H-89 also inhibited Alcian blue staining at day 5 (Fig. 4D). To examine the possibility of a cytotoxicity effect from H-89 on MPCs in micromass culture, we measured cell viability using trypan blue staining. Our results showed 93% of cells in both H-89 treated/untreated groups were unstained by trypan blue (Figure S1) indicating that H-89 did not increase cell death in our experiments. Together, these results indicate that activation of PKA signaling plays a predominant role in mediating PTH regulation of Runx1 and chondrogenesis in MPCs.

## Discussion

Although the effects of the method of PTH administration on osteogenic cells have been widely studied, recent evidence that teriparatide enhances chondrogenesis has also generated interest in its potential for articular cartilage repair. Recent animal studies have suggested that there are positive actions of intermittent PTH upon cartilage regeneration. Using a rabbit 5-mm-diameter full-thickness articular cartilage defect model, intermittent administration of PTH successfully induced the chondrogenic repair response [28]. Furthermore, systemic intermittent PTH treatment showed a chondro-regenerative effect in degenerating cartilage following a meniscal/ligamentous knee injury [29]. However, the molecular mechanism by which PTH promotes chondrogenesis remains unclear.

Cells isolated from E11.5 embryonic limb buds are mainly mesoderm mesenchymal progenitor cells, and can be differentiated into chondrocytes, osteoblasts or adipocytes in vitro. These cells quickly form chondrocyte nodules in micromass culture; therefore, the limb bud cell micromass culture system has been widely used to mimic condensation of the mexenchyme and the formation and maturation of the cartilage anlagen [30] during *in vivo* chondrogenesis. Alcian blue staining showed that chondrocyte nodules, one indication of cartilage formation, were detected as early as day 3 of micromass culture. This indicates that micromass culture provides an environment for rapid chondrogenesis of MPCs cultured *in vitro*. We then utilized this limb bud cell micromass culture system to study the mechanism underlying PTH-induced chondrogenesis. As expected, PTH-induced chondrocyte nodule formation further verified its anabolic effect during chondrogenesis. A positive correlation was observed between PTH1 receptor gene expression in MPCs and time in micromass culture [31], this finding generated speculation that the PTH-induced anabolic effect on MPCs may occur through interaction with the PTH 1 receptor. During the process of chondrogenic differentiation, *Sox9* is one of the early transcription factors synthesized by MPCs. In *Prx1Cre/Sox9* knockout mice, mesenchymal limb bud cells fail to undergo chondrogenic differentiation because the *Sox9* gene is inactivated before mesenchymal condensation occurs [32]. Although Sox9 plays a crucial role in MPCs and its expression is increased in micromass culture at day 3 and day 5 of PTH treatment, we did not see significant difference between PTH-treated and vehicle-treated cells at day 1. These data suggest *Sox9* might play a role in PTH-induced chondrogenesis, but not before day 3. In contrast, PTH significantly induced *Runx1* expression levels in limb bud cells as early as 6 hours after treatment, suggesting an essential role of Runx1 in PTH-mediated early phase chondrogenesis. Because a slight increase in Sox9 levels in response to PTH at day 3 and day 5 was observed, we believe this delayed increase may largely be due to a secondary effect from the earlier increase in *Runx1,* as *Sox9* was also reported as a target gene of *Runx1* during skeletal development and fracture healing [33]. Moreover, this increase in Sox9 may also result in further increases of Col2a1 and Col10a1 at days 5 and 7.

As previously mentioned, our *in vitro* studies have shown that Runx1 mediates mesenchymal stem cell commitment to the chondrogenic lineage [14], [34]. However, the role of *Runx1* in PTH-induced cartilage repair remains unknown. Since *Runx1* may be important at a stage of MPC differentiation to the chondrogenic phenotype that is prior to type II collagen expression, we performed *in vitro* deletions of *Runx1* in MPCs using shRNA lentivirus to examine whether *Runx1* is required for PTH-induced chondrocyte nodule formation. Alcian blue staining was performed at day 5 when PTH showed the highest effect on induction of chondrocyte nodule formation as compared to control. Our results show that *Runx1* inactivation in limb bud cells only slightly inhibited spontaneous chondrocyte nodule formation, but completely blocked PTH-induced chondrocyte nodule formation. These data clearly indicate that PTH regulates chondrogenesis of a MPC population in a *Runx1* dependent manner. Although *Sox9* is a possible target gene of *Runx1*, only a slight decrease in *Sox9* was caused by knocking down *Runx1* at this stage indicating that *Sox9* could also be regulated by other transcription factors.

Previous studies have suggested that PTH stimulates many osteogenic related pathways. Notably, activation of PKA signaling in response to a PTH-induced anabolic effect in bone is widely accepted. Activation of the PTH1R by intermittent PTH causes an acute activation of PKA and phosphorylation of transcription factors, such as CREB, which regulate transcription of Runx2 to promote osteoblast maturation [5], [35]. However, whether PKA signaling is also involved in PTH-induced *Runx1* expression in MPCs has not been characterized. Based on the fact that the PKA signaling pathway is also known to stimulate the expression of chondrocyte differentiation genes such as BMP2 [36], it seemed highly likely that PTH could also act in part by mediating PKA signaling in chondrogenesis. To further elucidate the relative contribution of PKA signaling to PTH-induced *Runx1* expression, PKA inhibitor H-89 was used in this study. When we treated limb bud MPCs with H-89, PTH-induced chondrogenesis and Runx1 expression were indeed severely impaired. Since no significant difference of p-PKA and Runx1 expression were observed between H-89 alone and co-treatment with PTH, we strongly believe that PKA activation is not only involved in PTH-induced chondrocyte nodule formation, but also is required for this process. However, the molecular mechanism by which PKA signaling directly regulates *Runx1* expression in MPCs is still unknown and needs further investigation. Since the possible contribution of other signaling pathways to Runx1 expression was not investigated in this study; we therefore cannot eliminate the possibility that *Runx1* could also be regulated by alternate or additional pathways.

## Conclusions

In conclusion, this study elaborates the effects of intermittent PTH treatment on chondrocyte nodule formation under chondrogenic conditions *in vitro*. Runx1 is required for chondrogenic differentiation of MPCs. PKA may support expression of Runx1 and thus promote chondrogenic differentiation in micromass. When either Runx1 or PKA are inhibited, MPCs lose the ability to undergo chondrogenic differentiation, and PTH is incapable of reversing this effect. These findings introduce a novel mechanism in which PTH promotes cartilage regeneration by regulating Runx1 expression, possibly through PKA signaling. This further supports the systemic intermittent PTH administration for the treatment of cartilage defects and osteoarthritis. Future study will focus on the identification of the PKA signaling binding sites on Runx1 promoter, which are responsible for PTH-induced Runx1 expression.

## Supporting Information

Figure S1 **Cell viability assay in micromass culture.** Limb bud cells in micromass culture were treated for 4 days with 10 µM PKA inhibitor H-89 and/or PTH 1–34 before harvested for trypan blue staining. Quantification of live cells over total cells showed no significant differences on cell viability between each group (n = 3, p>0.05).
(TIF)
